# A New Series of EDOT Based Co-Sensitizers for Enhanced Efficiency of Cocktail DSSC: A Comparative Study of Two Different Anchoring Groups

**DOI:** 10.3390/molecules24193554

**Published:** 2019-09-30

**Authors:** Ganesh Koyyada, Ramesh Kumar Chitumalla, Suresh Thogiti, Jae Hong Kim, Joonkyung Jang, Malapaka Chandrasekharam, Jae Hak Jung

**Affiliations:** 1 Department of Chemical Engineering, Yeungnam University, 214-1, Dae-hakro 280, Gyeongsan, Gyeongbuk 712-749, Korea; ganeshkoyyada@ynu.ac.kr (G.K.); sureshyu@ynu.ac.kr (S.T.); jaehkim@ynu.ac.kr (J.H.K.); 2 Department of Nanoenergy Engineering, Pusan National University, Busan 46241, Korea; rameshchitumalla@gmail.com (R.K.C.); jkjang@pusan.ac.kr (J.J.); 3 Inorganic and Physical Chemistry Division, CSIR-Indian Institute of Chemical Technology, Tarnaka, Hyderabad 500007, India; 4 Academy of Scientific & Innovative Research (AcSIR), CSIR-IICT, Tarnaka, Hyderabad 500007, India

**Keywords:** 3,4-ethylenediox-thiophene, dye-sensitized solar cells, co-sensitizer, power conversion effiecency, DFT

## Abstract

Herein, we report the design and synthesis strategy of a new class of five EDOT based co-sensitizers (CSGR1-5) by introducing different donors (2,3,4-trimethoxypheny, 2,4-dibutoxyphenyl, and 2,4-difluorophenyl) and anchoring groups (rhodamine-3-acetic acid and cyanoacetic acid) systematically. The synthesized metal-free organic co-sensitizers were employed for cocktail dye-sensitized solar cells along with N749 (black dye). The DSSC devices with a mixture of co-sensitizers (CSGR1-5) and N749 have shown a 7.95%, 8.40%, 7.81%, 6.56% and 6.99% power conversion efficiency (PCE) respectively, which was more than that of single N749 dye PCE (6.18%). Enhanced efficiency could be ascribed to the increased short circuit current (*J_sc_*) and open circuit voltage (*V_oc_*). The increased *J_sc_* was achieved due to enhanced light harvesting nature of N749 device upon co-sensitization with CSGR dyes and feasible energy levels of both the dyes. The *V_oc_* was improved due to better surface coverage which helps in decreasing the rate of recombination. The detailed optical and electrochemical properties were investigated and complimented with theoretical studies (DFT).

## 1. Introduction

Confronted with the massive challenges of future energy demand and environmental safety, scientific society and engineers are continuously struggling in finding and developing new energy resources. Towards finding a solution, the solar energy is recognized as the most reliable and promising candidate owing to its cleanliness, abundance and easy availability [1]. Recently, apart from silicon-based solar cells, different device techniques such as organic photovoltaic [2] Dye-sensitized solar cells [3], Perovskite Solar Cells [4], Copper Zink Tin Sulfide based solar cells (CZTS) [5], have achieved both scientific and commercial visibility, among which dye-sensitized solar cells (DSSC) was considered to be the most important device technique [3,6,7]. Since the publication of the first report on DSSC by O’Regan and Grätzal in 1991, the DSSCs have attracted significant attention due to their high-power conversion efficiency (PCE) of converting incident light to electricity and low-cost production [8]. Dye-sensitized solar cells (DSCs) could be effectively used in both outdoor and indoor applications. A great amount of research has been focused on n-type DSC, where a sensitized TiO_2_ electrode acts as photoanode, reaching an overall efficiency up to 14% [9,10,11], In a DSSC device, dye sensitizer plays an essential role in converting the solar energy to electrical energy by harvesting the solar light and injecting the excited electron into the TiO_2_ [12]. Wide absorption range of the solar spectrum, high molar extinction coefficient (ε_max_), and feasible energy levels are found to be the key parameters for achieving high efficiency [13,14]. Since the past decade, great efforts have been focused on the advancement of dye-sensitizers [7]. Mostly two different types of sensitizers, namely, metal complexes and metal-free sensitizers are being used as light harvesters in DSSC. N3, N719, and N749 dyes are some of the benchmark sensitizers in metal complex-based sensitizers for DSSC, which obtained more than 10% efficiency, because of their broad absorption range and high *J_sc_* value [15]. However, using ruthenium sensitizers for DSSC is not optimal because the molar extinction coefficient at higher wavelength region (λmax) is lower than that of organic dyes which limits the light harvesting capacity. Moreover, structural modifications on ruthenium dyes are limited because a smaller number of chelating ligands are compatible with the synthesis of various Ru complexes [16,17,18]. Despifigte a higher efficiency, ruthenium dyes suffer with degradation issues due to electrolyte compositions such as 4-tert-butylpyridine additive or nitrile based solvent molecules, which substitute the NCS ligand on ruthenium dyes at significant rate of increased temperature (80–100 °C) [17,19].

On the other hand, organic dyes can be modified to be able to absorb from the UV-visible range to near IR range (yellow [20] to red [21,22] to blue [23,24]) possessing high molar extinction coefficient than Ru dyes. Moreover, the D-π-A type of arrangement of organic sensitizers facilitates infinity range of potential structural modifications [25]. The majority of the reported organic dyes were synthesized based on triphenylamine, phenothiazine, phenoxazine, carbazole, boron dipyrromethane, squaraine and indolines [3,26,27,28,29], in which squarine, phthalocyanines, and cyanine sensitizers have shown more than 650 nm of maximum absorption wavelength (λmax) [30]. However, the DSSC performance of these metal-free organic dyes in terms of PCE was less than that of ruthenium-based dyes [16,18]. To harvest the full solar spectrum using single organic dye, one needs to introduce various groups, which would again complicate the synthesis and purification process—ultimately enhancing the device cost [31].

Co-sensitization technology is one of the best alternative approaches to fulfill the limitations of a single dye approach by sensitizing with a combination of two or more dyes in the DSSC device. For example, ruthenium dye has lower absorption power with a low molar extinction coefficient at a higher wavelength region, which would be complemented with the high molar extinction coefficient of organic dye, thereby imparting panchromatic nature and high PCE to DSSC device. [28,32] Earlier, numerous combinations have been reported for co-sensitization in DSSC such as Ruthenium dyes with organic dye and porphyrin with organic dye or metal-free organic dyes, which have shown an enhanced photovoltaic performance compared to that of single sensitizers. Yella et al., reported an enhanced efficiency for YD2-O-C8 porphyrin dye from 11.9% to 12.3% after a careful evaluation of DSSC in the process of co-sensitization using organic sensitizer. Moreover, porphyrins generally required difficult synthetic procedures with lower yields, notably, when anchoring and a redox group needs to be added at precise locations [33]. Apart from these, the co-sensitized DSSCs (cocktail DSSC) using ruthenium complex and metal-free dyes in the DSSC device have been extensively examined in the recent past. In our group, we have achieved a 11.4% certified record efficiency using a combination of N749 dye and Y1 organic dye [34]. It was mostly because of a change in the molecular size of the dyes, which was able to adequately adsorb on the surface of the TiO_2_ semiconductor, thereby enabling the device to harvest a larger number of incident photons from the light [35,36]. Inspired by these results various research groups, [35], in addition to our group [33,35,36,37,38], designed numerous organic co-sensitizers using high electron donors such as triphenylamine, indole, carbazole, phenothiazine, thiophene, etc. [39]. So far, very little research has been focused on small size co-sensitizers (such as Y1) possessing feasible optical and electrochemical properties with ruthenium complexes, which were known to be more advantageous than that of various organic dyes, because of their ability to adsorb into pores and gaps of TiO_2_ semiconductor surface, whereas the three dimensional ruthenium-based dyes cannot get adsorbed [7,40]. Moreover, in our group, we have reported a co-sensitized system of N749 and Y1 dyes by testing 1100 h of 1 sun light soaking at 55 °C, which showed remarkable enhancement in stability of the device by employing the Y1 as co-sensitizers [19].

**Figure 1:**
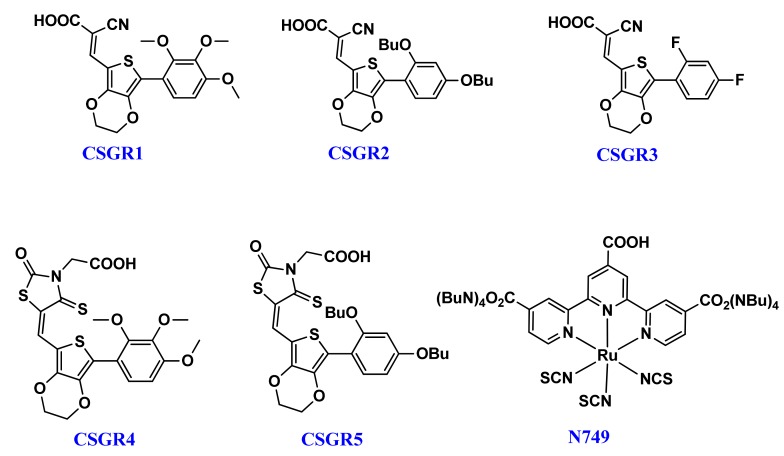
Chemical Structures of CSGR1-5 dyes.

3,4-ethylenediox-thiophene (EDOT) unit has been widely used as a conjugated bridge unit between the acceptor and donor groups of DSSC dyes, due to its capability to accelerate co-planarity and enhance the light harvesting capacity [41]. So far, 6% efficiency has been reported with EDOT based dyes, which were further extended to 8.2% by optimizing the anchoring group [30]. To carry forward the advantages of small organic co-sensitizers in cocktail DSSC and emphasize the role of EDOT central moiety, we have designed five small size new organic co-sensitizers coded as CSGR1-CSGRR5 in D-π-A manner. These five co-sensitizers (CSGR1-5), as shown in Figure 1, were synthesized by changing the donating groups (2,3,4-trimethoxypheny, 2,4-dibutoxyphenyl, 2,4-difluorophenyl) and anchoring groups (cyano-acrylic acid (CA) and rhodamine acetic acid (RA)) systematically and their DSSC performance as co-sensitizer with the N749 dye was examined. The final structure of CSGR1-5 dyes and N749 were shown in Figure 1.

## 2. Experimental Section

### 2.1. General

The 2,3-dihydrothieno[3,4-b][1,4]dioxine (EDOT), (2,3,4-trimethoxyphenyl)boronic acid (2,3,4-trimethoxyphenyl)boronic acid, (2,4-difluorophenyl)boronic acid, and (2,4-dibutoxyphenyl)boronic acid materials were bought from Sigma-Aldrich. The analytical grade solvents and chemicals were utilized for the synthesis and all the chemical reactions were carried out in the argon atmosphere. 60-120 mesh silica gel was used for chromatographic separations. ^1^H-NMR and C^13^-were performed on a Varian Mercury NMR 300 MHz spectrometers in CDCl_3_ or C_3_D_6_O solvent, where TMS was used as an internal reference. Absorption and emission spectra were recorded on an Agilent 8453 UV-vis spectrophotometer and Jobin-Yvon Horiba model fluorolog3 spectrometer, respectively. The cyclic (CV) or deferential pulses voltammograms (DPV) were acquired in DMF solution containing 0.3 mM dye and 0.1M tetrabutylammonium hexafluorophosphate (n-Bu_4_NPF_6_). The three electrodes consisted of Pt wire counter electrode and Ag/AgCl reference electrodes with a 50 mV/s scan rate.

### 2.2. Solar Cell Fabrication and Photovoltaic Characterization

The transparent conducting glasses (fluorine-doped tin oxide (FTO)) were cleaned carefully using ethanol, D.I water and acetone in an ultra-sonication process, respectively. The cleaned FTO glasses (Pilkington, 15 Ω/cm^2^) were coated with transparent TiO_2_ pastes (20–30 nm, Dyesol Ltd.) using the doctor blade method, followed by calcination for 30 min at 450 °C. The TiO_2_ scattering layer (200 nm, Dyesol Ltd.) consists of rutile TiO_2_, which was deposited on the transparent mesoporous TiO_2_ films, followed by sintering at 450 °C for 30 min. Two layers of TiO_2_ films were dipped in a 40 mM aqueous solution of TiCl_4_ at 70 °C for 30 min and then sintered at 450 °C for 30 min. The TiO_2_ thin films were immersed in the dye solution for 24 h in the dark at 25 °C. Where dye solution was prepared using 0.1 mM of CSGR dyes in THF and 0.2 mM N749 dye in tert-butanol and acetonitrile mixed solvent (1:1). After 24 h of soaking, the residual dye was rinsed with acetonitrile and dried. The platinum catalyst counter electrodes were prepared on FTO glasses using the doctor blade technique by H_2_PtCl_6_ solution, followed by pyrolysis at 450 °C for 30 min. The working electrode and Pt counter electrodes were assembled into a sealed sandwich cell with a 60 mm thick thermal adhesive film (Surlyn film, Dupont), which was then introduced with a liquid electrolyte solution containing 1-butyl-3-methylimidazolium iodide (0.7 M), lithium iodide (LiI, 0.2 M), iodine (I2, 0.05 M), and *t*-butylpyridine (TBP, 0.5 M) in acetonitrile/ valeronitrile (85:15, *v*/*v*) through pre-drilled two holes on the counter electrode.

The photo-current density-voltage (J-V) characteristics of synthesized dyes were measured under AM 1.5 irradiation with an irradiance of 100 mW/cm^2^ (PEC-L11, Peccell Technologies, Inc.). The incident monochromatic photon-to-current efficiencies (IPCEs) were measured using IPCE measurement instrument (PEC-S20, Peccell Technologies, Inc.) as a function of wavelength. Electrochemical impedance spectroscopy (EIS) was recorded using computer-controlled potentiostat (IVIUMSTAT, IVIUM) software at the open circuit voltage with a 10 mV of amplitude and an AC frequency range between 100 kHz and 0.1 Hz (PEC-L11, Peccell Technologies, Inc.)

### 2.3. Procedures for the Synthesis of CSGR Dyes

The detailed synthesis procedure for intermediate steps of CSGR dyes have been discussed supporting information.

(i) 4(E)-2-cyano-3-(7-(2,3,4-trimethoxyphenyl)-2,3-dihydrothieno[3,4-b][1,4]dioxin-5-yl)acrylic acid (CSGR1) [2,39].

A mixture of compound **3a** (0.120 g, 0.2 mmol), 2-cyanoacetic acid (0.056 g, 0.6 mmol), ammonium acetate (3 mg), and acetic acid (11 mL) was heated at 120 °C for 12 h. After cooling, water was added to quench the reaction. The precipitate was filtered and washed with water. The residue was purified by column chromatography on silica gel (CH_2_Cl_2_/methanol = 10/1, *v*/*v*) to yield CSGR1 as a red solid (118 mg, 88.5%). ^1^HNMR (300 MHz, CDCl_3_, δppm) 8.93 (s, 1H), 7.89 (d, 1H), 6.61 (d, 1H), 4.42 (m, 2H), 4.27 (m, 2H), 3.72(s, 3H), 3.69 (s, 3H), 3.61(s, 3H). ^13^C NMR (75 MHz, CDCl_3_, δppm ): 162.7, 156.3, 146.5, 145.4, 139.8, 134.1, 130.9, 127.9, 114.7, 112.5, 103.1, 64.6, 62.2, 56.3. ESI-MS (C_19_H_17_NO_7_S): calculated: 403.07, found: 402.06 (M−H). CHNS analysis calculated for C_19_H_17_NO_7_S C: 56.57, H: 4.25, N: 3.47, S: 7.95, found: C: 56.42, H: 4.11, N: 3.39, S: 7.83

(ii) (E)-2-cyano-3-(7-(2,4-dibutoxyphenyl)-2,3-dihydrothieno[3,4-b][1,4]dioxin-5-yl)acrylic acid (CSGR2).

Dye CSGR2 was synthesized by employing the same procedure as that for CSGR1 to yield red solid (0.108 mg, 81%) ^1^HNMR (300 MHz, CDCl_3_, δppm) 8.96 (s, 1H), 7.61 (d, 1H), 6.98 (d, 1H), 6.62 (m, 1H), 4.47 (m, 2H), 4.37–4.29 (m, 6 H), 1.83 (m, 4H), 1.41 (m, 4H), 0.93 (t, 6H). ^13^C NMR (75 MHz, CDCl_3_, δppm): 163.1, 161.8, 155.6, 145.9, 144.5, 133.1, 131.8, 128.2, 114.7, 103.1, 99.5, 68.8, 67.6, 64.3, 31.4, 19.7, 14.2. ESI-MS: calcd 457.16, found: 356.14 (M−H). CHNS analysis calculated for C_24_H_27_NO_6_S C: 63.00, H: 5.95, N: 3.06, S: 7.01, found: C: 62.97, H: 5.91, N: 2.98, S: 6.93.

(iii) (E)-2-cyano-3-(7-(2,4-difluorophenyl)-2,3-dihydrothieno[3,4-b][1,4]dioxin-5-yl)acrylic acid (CSGR3).

Dye CSGR3 was synthesized by employing the same procedure as that for CSGR1 to yield red solid (0.108 mg, 81%) ^1^HNMR (300 MHz, CDCl_3_, δppm) 8.93 (s, 1H), 7.78 (d, 1H), 7.31 (m, 1H), 7.11 (d, 1H), 4.56 (m, 2H), 4.41 (m, 2H). ^13^C NMR (75 MHz, CDCl_3_, δppm): 163.8, 162.3, 160.2, 145.9, 144.2, 133.0, 130.6, 127.7, 114.9, 104.7, 103.1, 64.6. ESI-MS: calculated: 349.02, found: 348.01 (M -H) CHNS analysis calculated for C_16_H_9_F_2_NO_4_S C: 55.01, H: 2.60, N: 4.01, S: 9.18, found: C: 54.97, H: 2.51, N: 3.88, S: 9.07.

(iv) (E)-2-(2-oxo-4-thioxo-5-((7-(2,3,4-trimethoxyphenyl)-2,3-dihydrothieno[3,4-b][1,4]dioxin-5-yl)methylene)thiazolidin-3-yl)acetic acid (CSGR4).

The CSGR4 was synthesized by following the procedure used for the synthesis of CSGR1, except the use of rhodanine-3-acetic acid (0.036 g, 0.376 mmol) anchoring group instead of 2-cyanoacetic acid to afford CSGR4 (0.115 g, 86%) as a dark yellow solid. ^1^HNMR (300 MHz, CDCl_3_, δppm): 8.13 (s, 1H), 7.90 (d, 1H), 6.85 (d, 1H), 4.71 (m, 2H), 4.45 (m, 4H). ^13^C NMR (75 MHz, CDCl_3_, δppm): 196.2, 170.7, 169.6, 156.1, 144.9, 141.2, 133.5, 131.1, 128.6, 119.3, 113.1, 64.5, 60.9, 56.4, 51.8. ESI-MS: calculated: 509.03, found: 508.02 (M -H). CHNS analysis calculated for C_21_H_19_NO_8_S_3_ C: 49.50, H: 3.76, N: 2.75, S: 18.88, found: C: 49.39, H: 3.65, N: 2.61, S: 18.79.

(v) (E)-2-(5-((7-(2,4-dibutoxyphenyl)-2,3-dihydrothieno[3,4-b][1,4]dioxin-5-yl)methylene)-2-oxo-4-thioxothiazolidin-3-yl)acetic acid (CSGR5).

The CSGR4 was synthesized by following the procedure used for the synthesis of CSGR1, except the use of rhodanine-3-acetic acid (0.036 g, 0.376 mmol) anchoring group instead of 2-cyanoacetic acid to afford CSGR5 (0.121 g, 87%) as a dark yellow solid. ^1^HNMR (300 MHz, CDCl_3_, δppm): 8.10 (d, 1H), 7.48 (s, 1H), 6.86 (m, 1H), 6.62 (s, 1H), 4.63 (m, 2H), 4.28-4.21 (m, 6H). ^13^C NMR (75 MHz, CDCl_3_, δppm): 196.2, 171.5, 170.2, 162.3, 155.7, 144.6, 133.1, 129.9, 127.7, 119.6, 114.7, 99.8, 69.3, 64.6, 52.2, 31.5, 19.4, 13.9. ESI-MS: calculated: 563.11, found: 562.11 (M−H). CHNS analysis calculated for C_26_H2_9_NO_7_S_3_ C: 55.40, H: 5.19, N: 2.48, S: 17.18, found: C: 55.37, H: 5.13, N: 2.36, S: 16.88.

## 3. Results and Discussion

### 3.1. Synthesis

We have synthesized five organic molecules in D-π-A manner, by systematically replacing them with the substituted benzenes as donors and the CA and RA as acceptors on ethylene dioxythiophene center core moiety. All the sensitizers were well characterized with NMR and MASS spectroscopy.

**Figure 2:**
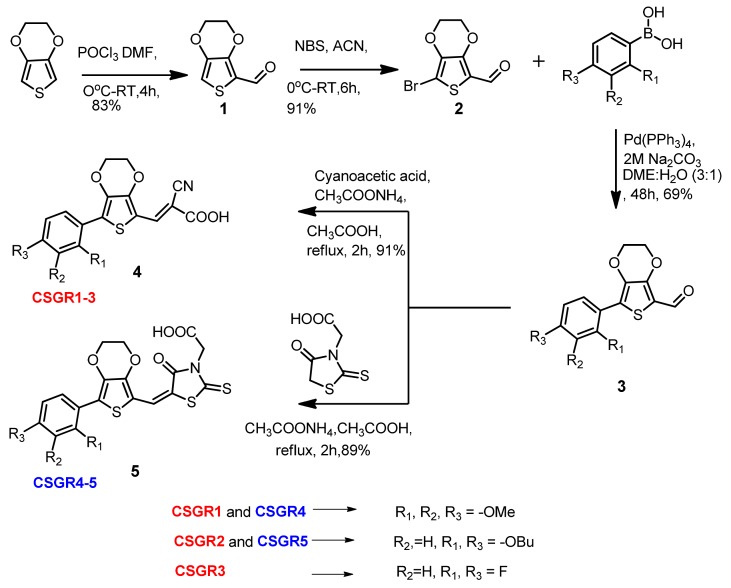
Synthesis of CSGR1-5 Sensitizers.

The reaction path and conditions for the synthesis of CSGR1-5 sensitizers were shown in Figure 2. The commercially accessible material 3,4-Ethylenedioxythiophene was subjected to Vilsmeier-Haak Formylation reaction to acquire the intermediate Compound **1** with a 81% yield. Compound **1** was brominated using N-Bromosuccinimide to obtain Compound **2** with a 64% yield. The desired compounds (**3a**–**3d**) were accomplished by condensation reaction via Suzuki coupling with corresponding boronic acids, (2,3,4-trimethoxyphenyl)boronic acids, (2,4-dibutoxyphenyl)boronic acid and (2,4-difluorophenyl)boronic acid with a 60% to 69% yield. The final compounds (CSGR1-5) were synthesized from the **3a**–**3d** by following Knovanagal condensation with CA or RA anchoring groups using NH_4_OAC and acetic acid with good yields (89% to 91%).

### 3.2. Optical and Electrochemical Measurements

**Figure 3:**
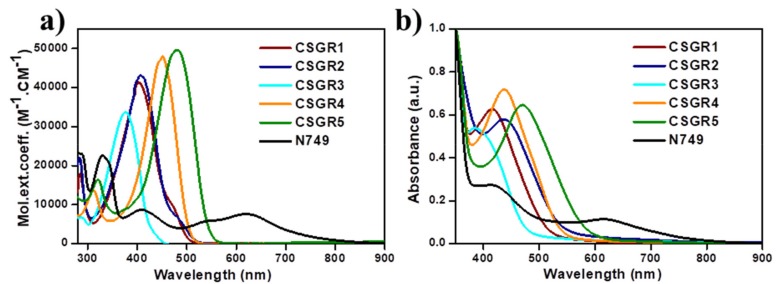
(**a**) UV-visible Spectral analysis of CSGR1-5 in DMF solution and (**b**) on TiO_2_ thin film.

The electronic absorption spectra of co-sensitizers (CSGR1-5) in N,N-Dimethylformamide (DMF) were shown in Figure 3a, and the resultant data were summarized in Table 1. CSGR1-5 dyes displayed two major bands in the Figure 3a, the band present at the lower wavelength region corresponds to the π-π* transitions localized between auxiliary donors and EDOT center moiety and another band present at the higher wavelength region (300–550) corresponds to the charge transfer (CT) between EDOT and the CA or RA anchoring groups [42]. The wavelength maximum (λmax) of the CSGR1-5 dyes in DMF solution was 402, 408, 376, 450 and 482 nm, respectively. All the CSGR dyes have shown higher molar extinction co-efficient (ε_max_) than N749 (0.8725 × 10^4^ M^−1^ cm^−1^) at 300 to 550 regions i.e., 4.3904, 4.1182, 3.3557, 4.7688 and 4.9522 × 10^4^ M^−1^ cm^−1^. It is worth mentioning that the high ε_max_ demonstrated by these co-sensitizers (CSGR dyes) at CT transition range was beneficial for harvesting maximum number of photons from incident light at a particular wavelength region by a minimum amount of dye loading so that it could facilitate enough space on the semiconductor (TiO_2_) for binding of other dye molecules. Among CA and RA based dyes, high absorption property was observed for RA based dyes (25–80 nm), which was attributed to the enhanced π-conjugation due to 4-oxo-2-thioxothiazolidine ring in RA anchoring group. Among 2,3,4-trimethoxybenzene (CSGR1 and CSGR4) and 2,4-dibutoxybenzene (CSGR2 and CSGR5) based dyes, a high absorption nature was observed for 2,4-dibutoxybenzene based dyes, which indicates the low donating nature of 2,4-dibutoxybenzene donating group than that of 2,4-dibutoxybenzene.

Figure 3b illustrates the absorption spectra of the CSGR dyes on TiO_2_ thin films in comparison with N749 dye. The TiO_2_ film sensitized with CSGR dyes has displayed a λmax of 415, 437, 389, 436 and 471 nm respectively, which were found to be a red shift of CSGR1-3 dyes by 13, 29 and 13 nm upon adsorption on TiO_2_. Generally, this type of red shifted absorption spectra on TiO_2_ compared to the solution state absorption spectra have been observed for many dyes in organic, which could be attributed to the J-aggregation that benefits the capture of photons from an extended region [13] Whereas CSGR5 and 6 have shown blue shifted absorption spectra of about 14 and 11 nm, which indicates the low energy transfer with TiO_2_.

**Figure 4:**
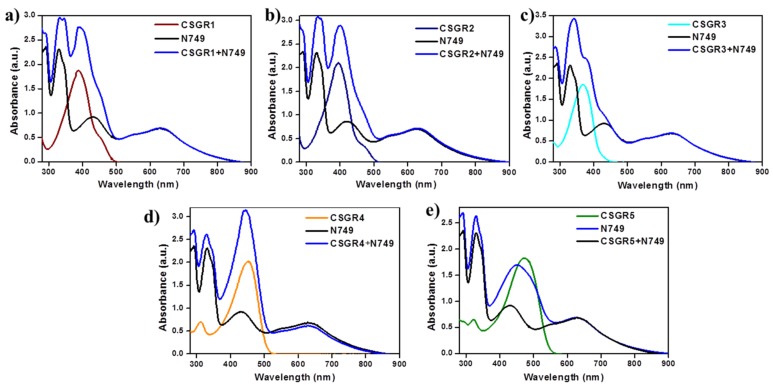
(**a–e**) are comparative absorption spectra of CSGR (0.05 mm), N749 (0.1 mm) and mixture of N749 and CSGR1-5 dyes (0.1 and 0.05 mm) in DMF.

**Figure 5:**
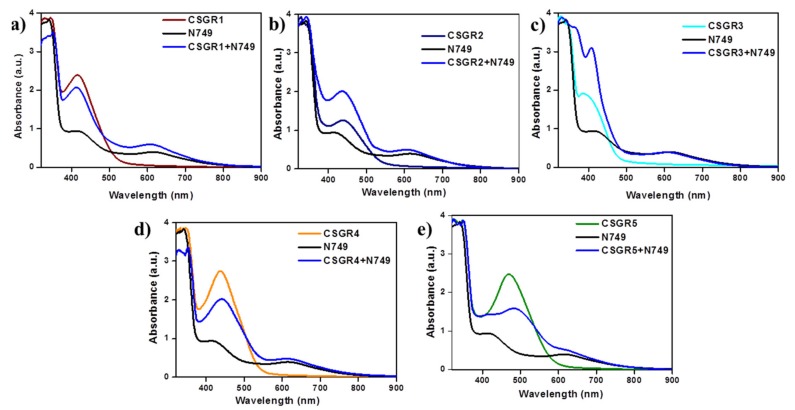
(**a–e**) are comparative absorption spectra of CSGR (0.3 mm), N749 (0.3 mm) and mixture of N749 and CSGR dyes (0.2 and 0.1 mm) on TiO_2_.

In order to understand the effect of individual dye on overall light absorption profile of cocktail dyes, we have performed the absorption spectra of cocktail dyes in solution state and on TiO_2_ thin film, which were displayed in Figure 4 and Figure 5 by comparing with their individual absorption properties. In the solution state, absorption spectra displayed enhanced absorbance profile for the cocktail dye solution in the range 300–550 nm compared to their corresponding individual dyes, which could be attributed to the strong molar extinction co-efficient of EDOT based CSGR dyes. Similarly, the same enhanced absorption profile was observed when absorption spectra were analyzed on TiO_2_. These results indicate that the cocktail dye system of N749 and CSGR dyes shows better light harvesting efficiency than that of individual N749 dye in this wavelength region.

The emission spectra of CSGR dyes were measured in the DMF solvent upon excitation at their λ_max_, and corresponding curves and data were shown in Figure S1 and Table 1. Figure S1 shows the absorption fluorescence intercept plots, which were used for calculating their bandgap and stock shift values. The observed large stock shift values (80–120 nm) suggests the efficient intramolecular charge transfer [43].

**Figure 6:**
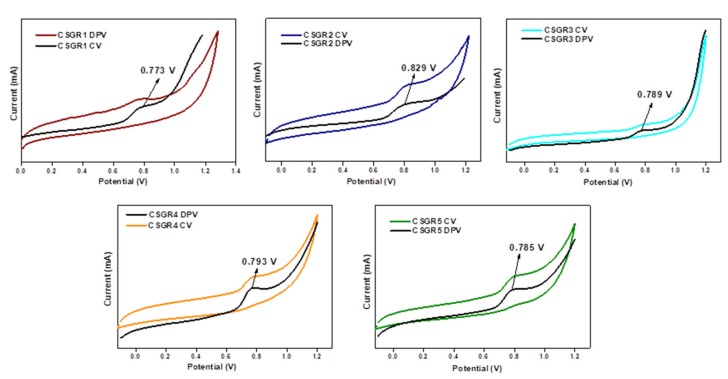
CV and DPV voltammograms of CSGR1-5 dyes in DMF.

The CV was performed to ensure the electrochemical nature and evaluate positions of the ground state oxidation potential [GSOP (E_ox_)] and excited state oxidation potentials [ESOP (E_ox_*)] of the CSGR dyes. CV and DPV were executed for CSGR dyes using DMF solution of 0.1 M TBAPF_6_ support electrolytes. The corresponding curves and data were presented in Figure 6 and Table 1. The analyzed GSOP of CSGR1-5 dyes were 0.773, 0.829, 0.789, 0.793, and 0.785 V (versus NHE) respectively, Here, onset oxidation potentials of oxidation peak of CV and DPV were used to calculate the GSOP, which were highly positive enough than that of redox electrolyte (I^−^/I_3_^−^), i.e., 0.4 V (−5.2 eV) versus NHE [43] for efficient dye regeneration. The ESOP (*E*_ox_*) of CSGR1-5 dyes were 1.827, 1.751, −2.021, −1.535 and 1.717 V, respectively, which were obtained by subtracting *E*_0-0_ from *E*_ox_ (*E*_ox_ – *E*_0-0_). Here E_0-0_ of CSGR dyes was measured from the intercept plots of absorption and fluorescence (Figure 5) by using the following formula:

(1) $$E_{0-0}=\frac{1240}{\lambda }$$

**Figure 7:**
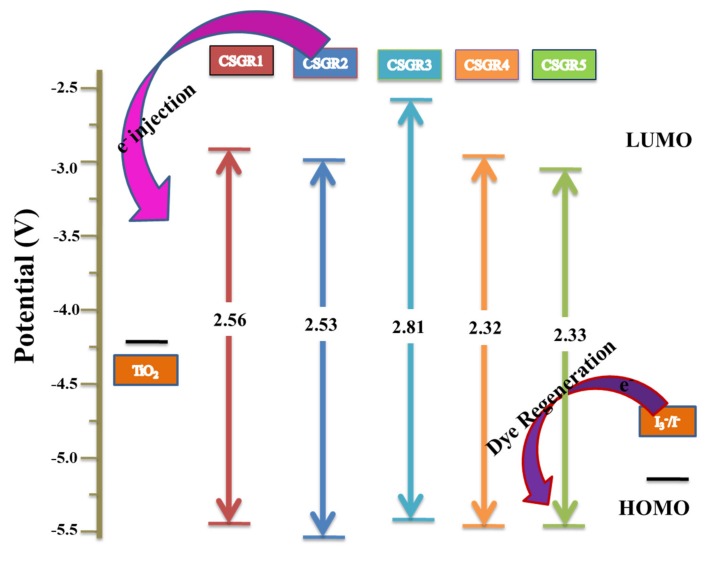
The schematic energy level diagram for CSGR1-5 based on UV-visible absorption and electrochemical data.

Here, wavelength (*λ*) is considered from the absorption and emission intercept point. The obtained *E*_0-0_ values of CSGR dye were 2.56, 2.53, 2.81, 2.55 and 2.33 eV, respectively. The ESOP (*E*_ox_^*^) of CSGR1-5 dyes were highly negative than that of TiO_2_ semiconductor conduction band, i.e., ~0.5 V versus NHE (−4.2 eV), facilitating adequate electron injection. The schematic energy level diagram for CSGR dyes was constructed based on the obtained results as shown in Figure 7.

### 3.3. Computational Studies

To explore the structural, electrochemical, and photophysical properties of the CSGR1 to CSGR5 dyes, we have carried out a thorough computational investigation. All the reported calculations have been performed by Gaussian 16 quantum chemistry software [44]. The ground state geometries of the dyes were optimized using a hybrid B3LYP [45,46] functional in combination with the 6-31G(d) basis set. The minima of the structures on the potential energy surface were confirmed from the vibrational frequency analysis with no imaginary frequencies. The optimized geometries were then subjected to time-dependent density functional theory (TDDFT) simulations to obtain UV-visible absorption spectra of the dyes. The photophysical properties of the dyes for the first 25 excited states were simulated in DMF solvent. The solvent effects in simulations were considered using the polarizable continuum model [47,48] as implemented in Gaussian 16. The TDDFT simulations were performed using TD-CAM-B3LYP function at the same level of a theory similar to that used for the ground state optimization of the dyes.

**Figure 8:**
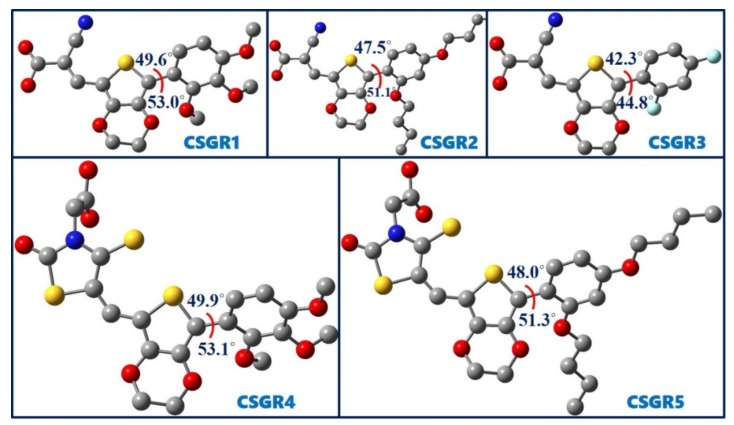
Optimized ground state geometries of the dyes CSGR1 to CSGR5 obtained at B3LYP/6-31G(d) level of theory.

Figure 8 shows the optimized geometries of five dyes, CSGR1 to CSGR5 along with the dihedral angles between thiophene and attached phenyl ring. The observed dihedral angles were found to be around 50°. The substitutions present on the phenyl ring disturbed the planarity of the dye molecule. The nonplanar structure of the dye prevents the dye aggregation on TiO_2,_ thereby resulting in the improved power conversion efficiency.

**Figure 9:**
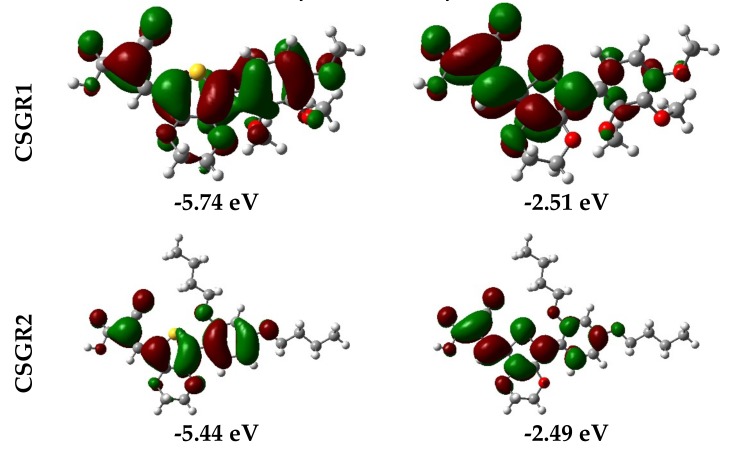

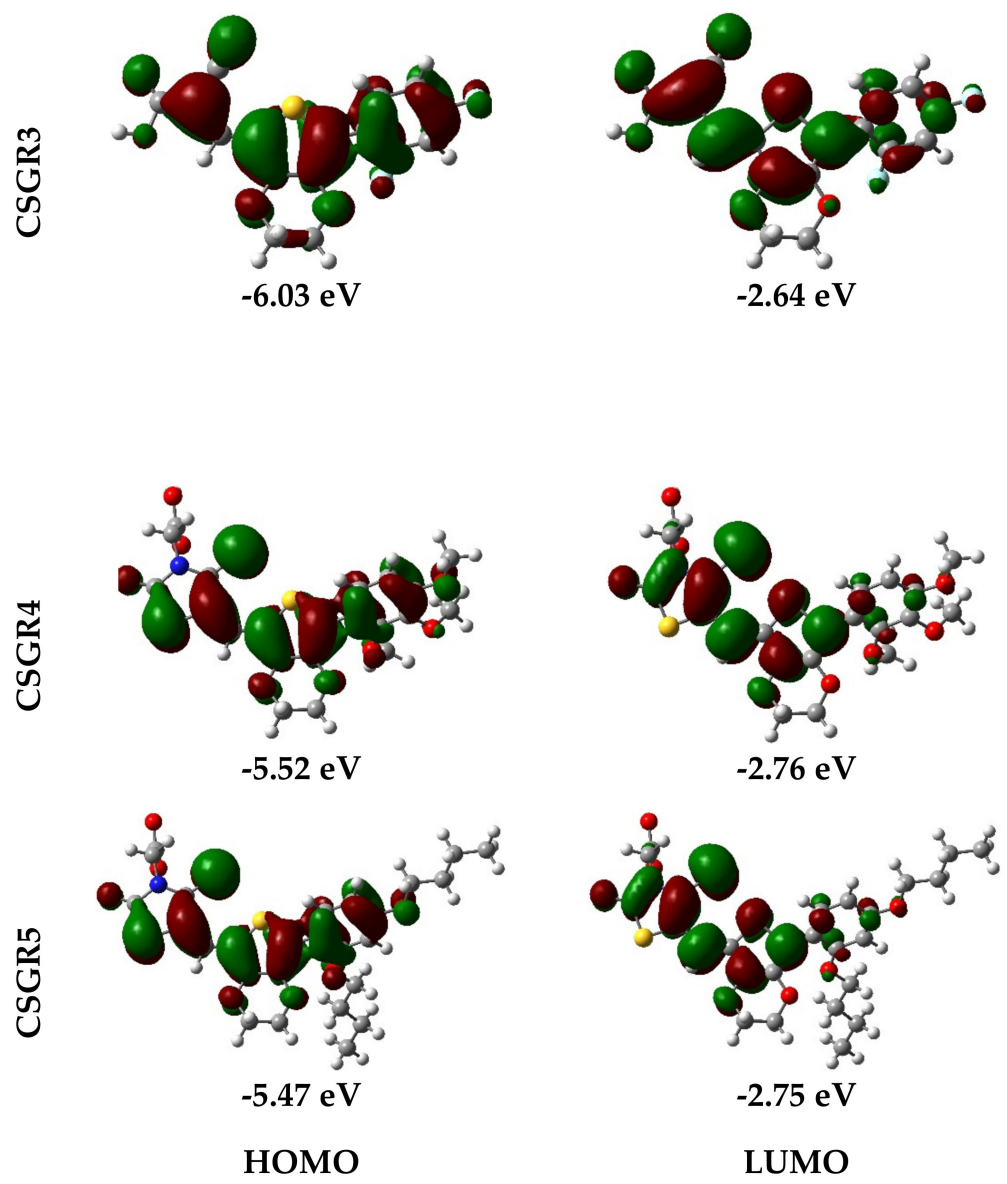
Electron density distribution in frontier molecular orbitals of the dyes CSGR1 to CSGR5 and the corresponding eigenvalues.

Electron density distribution plots of the dyes were obtained from the population analysis and their frontier molecular orbitals along with their eigenvalues, as shown in Figure 9. The electron density in HOMO was mainly located on the EDOT moiety and shifted towards the anchoring group in LUMO. From Figure 8, it can be observed that the electron density transfer towards the anchoring group was more in case of cyanoacetic acid (CA) based dyes over rhodamine acetic (RA) acid-based dyes. The low electron density shift in RA anchoring dyes was due to the presence of a methylene group between -COOH and EDOT moiety, which lowers the charge transfer. The low power conversion efficiency exhibited by RA-based dyes over CA-based dyes could be attributed to their low electron transfer to the anchoring group, thereby leading to poor electron injection to the TiO_2_ conduction band. The theoretically obtained HOMO and LUMO values were in good agreement with the experimental data obtained from the cyclic voltammetry.

**Figure 10:**
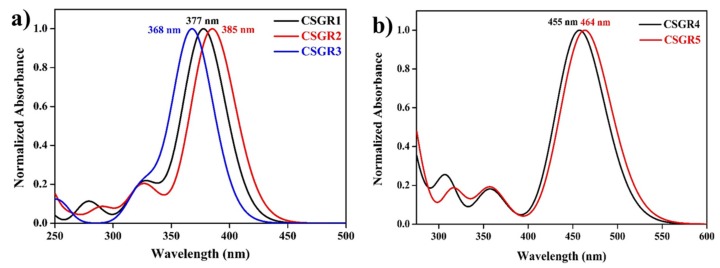
Simulated UV-visible absorption spectra of the dyes CSGR1 to CSGR5 obtained in DMF solvent at TD-CAM-B3LYP/6-31G(d) level of theory.

The optical properties of the dyes were obtained using TDDFT formalism, and the simulated absorption spectra were shown in Figure 10. The theoretically calculated absorption spectra reproduced the absorption bands that were observed in the experimental UV-visible absorption spectra. CSGR1 to CSGR3 dyes have shown intense absorption maxima around 375 nm, whereas, CSGR4 and CSGR5 dyes have shown intense absorption maxima around 460 nm. Compared to the absorption of the dye CSGR1, the dyes CSGR2 and CSGR3 have shown 8 nm bathochromic and 9 nm hypochromic shifts, respectively. Among CSGR4 and CSGR5 dyes, CSGR5 has shown 9 nm bathochromic shifts compared to that of CSGR4. These intense absorption peaks originated mainly from the HOMO → LUMO excitation. The observed shifts in the experimental absorption spectra were reproduced from the simulations.

### 3.4. Photovoltaic Properties

**Figure 11:**
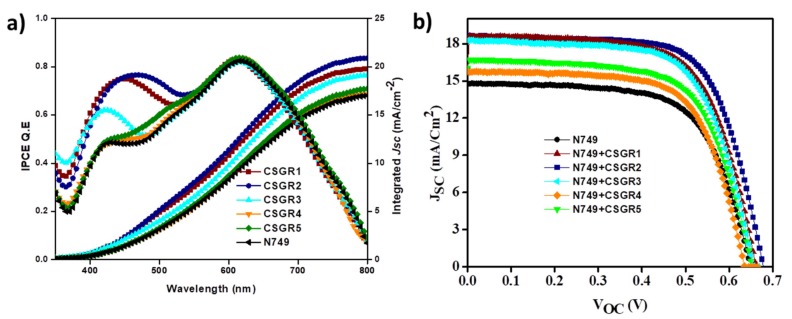
(**a**) IPCE and integrated current density plots and (**b**) are the *J*-*V* plots of DSSCs based on N749 dye with CSGR1-5 dyes.

The photovoltaic performances of the co-sensitized DSSC devices (cocktail DSSC) were examined to establish a relationship between structures of co-sensitizer and their performances with ruthenium dyes. The addition of varying auxiliary substituents and anchoring groups (CA and RA) on the principal moiety of EDOT have shown to have a profound influence on the incident photon-to-current conversion efficiency (IPCE) and photovoltaic properties of the cocktail DSSC devices. The photovoltaic properties of the DSSC devices sensitized with N749 and co-sensitizers (CSGR1-5 dyes) on a TiO_2_ semiconductor electrode have been analyzed in standard AM 1.5 irradiations (100 mW/cm^−2^). The resultant current-voltage (*J*-*V*) plots have been presented in Figure 11b and the related data was summarized in Table 2. By using CSGR dyes as co-sensitizers instead of N749 dye alone, the photovoltaic performance of DSSC was enhanced as observed in J-V curves and Table 2. The overall conversion efficiency (ɳ) of N749 alone was 6.18, whereas by using EDOT co-sensitizers (CSGR1-5) the efficiency has been enhanced to 7.95%, 8.40%, 7.81%, 6.56% and 6.99% respectively. The observed enhanced efficiency could be attributed to the improved photovoltaic parameters i.e., short-circuit current density (*J_sc_*), open-circuit voltage (*V_oc_*) and fill factor (FF). The *J_sc_* and *V_oc_* of N749 alone were 14.73 mA cm^−2^ and 0.648 V, whereas after employing CSGR1-5 co-sensitizers the *J_sc_* was enhanced to 18.51, 18.55, 18.15, 15.72, and 16.59 mA cm^-2^, respectively, and the *V_oc_* was enhanced to 0.659, 0.674, 0.658, 0.634 and 0.651 V, respectively. The enhanced *J_sc_* might be ascribed to the increased light harvesting nature at 300–500 nm regions due to the addition of CSGR co-sensitizers, which possess a high molar absorption coefficient. The observed enhanced *V_oc_* of cocktail DSSC devices could be attributed to the decreased recombination rate between the injected electrons in the conduction band of TiO_2_ semiconductor and I_3_^-^ species in the redox electrolyte. Owing to their small size, the co-sensitizers provide better surface coverage [19,34,37] by getting adsorbed into the pores and gaps on TiO_2_, whereas the ruthenium-based dye molecules cannot get absorbed (discussed in detail in dye loading section). Though RA based co-sensitizers have shown high absorption properties in DMF solvent than CA based dyes, it could not be transformed into high efficiency. As mentioned by Weihong Zhu et al. [49], this might be due to the methylene group present between EDOT center moiety and carboxylic acid, which would prevent efficient electron injection into the conduction band of TiO_2_ by breaking the conjugation. Therefore, the resulting unfavourable distribution of LUMO may possibly lead to low PCE for RA based dyes. Among all the co-sensitizers, CSGR2 and N749 based cocktail DSSC device has shown highest *J_sc_*, *V_oc_*, and efficiency due to high light harvesting nature of CSGR2.

The IPCE for the DSSC sensitized with N749 and cocktail DSSC devices (co-sensitizers and N749) have been measured and plotted in Figure 11a. All the DSSC devices have shown broad coverage of IPCE spectra from visible range to near IR range. The DSSC devices sensitized with N749 and CSGR1-CSGR3 have shown impressive IPCE in the 300 to 500 region, compared to N749 alone, with an unfavourable 72%, 73% and 60%, respectively. This might be because of the high molar extinction coefficient of CSGR1-CSGR3 dyes than that of the I3- (2.5 × 104 m^−1^ cm^−1^) at this particular wavelength. Therefore, these co-sensitizers reduced the competitive absorption with electrolyte resulting in the enhancement of IPCE [50], whereas RA based co-sensitizers failed to enhance the IPCE as expected because of the low electron injection efficiency due to the methylene group and unfavourable LUMO distribution [49]. According to the IPCE spectra, the integrated current density of CSGR and N749 dyes were 19.76, 20.91, 19.12, 17.16, 17.70 and 17.01, mA/cm^2^, respectively. The obtained *Jsc* values trend has matched with their respective *J-V* characteristics, within the error range.

**Figure 12:**
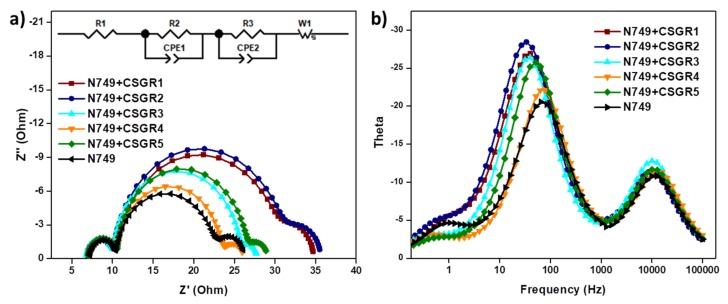
(**a**) EIS Nyquist plots of cocktail DSSC devices of N749 and CSGR dyes measured under a dark condition. (**b**) Bode-Phase plots for cocktail DSSC devices of N749 and CSGR dyes.

To further understand the recombination rate and charge recombination lifetime in the DSSC device by employing CSGR1-5 co-sensitizers along with N749, the electrochemical impedance spectroscopy (EIS) was executed under dark condition. The Nyquist and bode plots for N749 and co-sensitized devices of CSGR1-5 were shown in Figure 12a,b, and the equivalent circuit fitting results were summarized in Table 3. In the EIS Nyquist plots, three semicircles were observed, the first and small semicircle at higher frequency region corresponds to the charge transfer process (R2) at the counter electrode and electrolyte interfaces. The large or mid-frequency region signifies the recombination resistance (R3) at the working electrode of the TiO_2_/dye/electrolyte interface [39,49]. The third semicircle (low-frequency range) indicates the Warburg diffusion process of I^−^/I_3_^−^. The obtained recombination resistance values (R3) in dark impedance analysis of DSSC devices with N749 and CSGR dyes were 19.38, 20.37, 15.28, 12.6, and 15.64 Ω, respectively. As a matter of fact, the higher the R3 values, the lower the charge recombination rate between the conduction band of TiO_2_ semiconductor and the electrolyte will be. The R3 values of cocktail DSSC devices with N749 and CSGR dyes decreased in the order of CSGR2 > CSGR1 > CSGR3 > CSGR5 > CSGR4 > N749. The obtained R3 values were consistent with the *V_oc_* values obtained in J-V curves. Among all the CA and RA based co-sensitizers, the dye with the 2,4-dibutoxyphenyl group showed higher R3 values, which indicates that the introduction of the dibutoxy group on the donor group of EDOT dyes has greatly enhanced the charge recombination resistance compared to other donating groups in CSGR dyes. Furthermore, the Bode phase plots (Figure 12b) were analyzed to determine the injected electron recombination lifetime of eTiO_2_ by following the relation, Ʈ_CB_ = 1/2πƒ in which Ʈ_CB_ is electron lifetime in TiO_2_ and ƒ is the peak frequency from bode plots. The electron lifetime for the CSGR co-sensitized DSSC devices and N749 dye was found to be 4.46, 4.75, 3.89, 2.43, 3.08 and 2.17 ms, respectively. The observed trend of the recombination lifetime was in par with the EIS results and *V_OC._* These results clearly portray the advantage of using co-sensitizers in obtaining decreased recombination rate and increased recombination life time owing to their small size and strong anchoring groups [40,51].

### 3.5. Dye Loading Analysis

In order to quantify the dye loading on the co-sensitized TiO_2_ thin film, adsorption-desorption studies were carried out for CSGR and N749 dyes both individually and in combination as well. Initially, the individually sensitized TiO_2_ films were dipped into 0.02 M NaOH solution, and the approximate amount of dye loading was calculated by analysing the absorption spectra of NaOH solution by monitoring the peak at their λmax [42]. The dye loading amount of CSGR dyes were 4.17 × 10^−7^, 4.21 × 10^−7^, 3.92 × 10^−7^, 3.11 × 10^−7^ and 3.22 × 10^−7^ mol cm^−2^, respectively, while N749 has 2.45 × 10^−^ mol cm^−2^. The total dye loading of co-sensitized photoanode was 3.43 × 10^−7^, 3.51 × 10^−7^, 3.28 × 10^−7^, 2.92 × 10^−7^, 3.09 × 10^−7^ mol. These results could be explained by the differences in molecular sizes and anchoring groups of N749 and CSGR dyes. Increased dye loading was accomplished by the adsorption of larger size N749 molecules onto TiO_2_ initially followed by the adsorption of smaller size CSGR molecules in such a way that it would fill the gaps between larger N749 molecules in the process of sensitization. In comparison to N749 molecules, CSGR dyes exhibit higher binding capacity to the TiO_2_ surface owing to their small size and strong anchoring group [51]. The back–electron recombination with the electrolyte or dye molecules was further prevented by the formation of a blocking layer covering the complete TiO_2_ nanoparticle due to increased dye loading [42,51]. This eventually led to decreased recombinations and increased *V_oc_* values, as evidently observed in case of experimentally co-sensitized DSSC.

## 4. Conclusions

In summary, a new series of EDOT based co-sensitizers (CSGR1-5) have been synthesized and explored along with the N749 for cocktail DSSC, and their relative performances were compared. The efficiencies were significantly enhanced from 6.18% to 7.95%, 8.40%, 7.81%, 6.99% and 6.56%, respectively. The corresponding enhancement in the efficiencies could be attributed to the increased IPCE at 300–550 nm regions, possibly due to the complementary absorption property of the co-sensitizers with the electrolyte and N719 dye, thereby harvesting a larger number of photons at this region, which in turn enhanced the *J_sc_* and PCE. Moreover, improved *V_oc_* values were observed for cocktail DSSC devices than N749 based dyes, most likely due to the small size of co-sensitizers, which may occupy the pores and gaps between the three-dimensional ruthenium-based dyes resulting in the suppression of back electron recombination by preventing the interaction between TiO_2_ semiconductor and I_3_^-^ electrolyte ions—evidently observed in EIS analysis. Among the co-sensitizers, RA based dyes have shown low PCE than CA-based dyes, which might be ascribed to the low electron injection efficiency due to the presence of methylene group between the anchoring group and EDOT center moiety of RA based dyes. This eventually led to the unfavourable LUMO distribution—evidently observed from the electron density distribution plots (Figure 9). Among CA based dyes, observed PCE performance decreased in the order of CSGR2 > CSGR1 > CSGR3, which depicted the donating nature of auxiliary groups (2,4-dibutoxyphenyl, 2,3,4-trimethoxyphenyl, and 2,4-difluorophenyl).

## Figures and Tables

**Table 1:** Photophysical and electrochemical data of CSGR1-5 dyes.

Dye	*λ*max (Solution) (nm) ^a^	*Ε* (M^−1^ Cm^−1^)	*λ*max (TiO_2_) (nm) ^b^	*λ*em (nm) ^c^	Stokes Shif (nm)	*E*_0X_ (V) ^d^	*E*_0-0_ (V) ^e^	*E*_0X_* (V) ^f^	HOMO (eV) ^g^	LUMO (eV) ^h^
CSGR1	402	41182	415	526	101	0.773	2.56	−1.787	5.473	−2.913
CSGR2	408	43904	437	533	114	0.829	2.53	−1.701	5.529	−2.993
CSGR3	376	33557	389	478	85	0.789	2.81	−2.021	5.489	−2.679
CSGR4	450	47688	436	528	92	0.793	2.55	−1.757	5.493	−2.943
CSGR5	482	49522	471	574	78	0.785	2.33	−1.545	5.485	
N749	623	8725	618							

^a^ Absorption spectra were measured in DMF. ^b^ Absorption spectra were measured on TiO_2_ thin film. ^c^ Emission in DMF. ^d^ Oxidation potentials (E_0X_) were measured using CV and DPV method from onsite potentials. ^e^ The bandgap (E_0-0_), was obtained from the UV-visible and emission intercept plots. ^f^ *E*_OX_*= (*E*ox − *E*_0-0_). ^g^ HOMO = −e[*E*ox (V) + 4.7 ev]. ^h^LUMO = −e[(*E*ox (v) + 4.7) − *E*_0-0_] eV.

**Table 2:** Photovoltaic measurement data of fabricated DSSC devices.

Dye	*J_SC_* (mA cm^−2^)	*V_OC_* (V)	FF	*η* (%)
N749	**14.73**	**0.648**	**64.74**	**6.18**
	14.32 + 0.41	0.637 + 0.008	64.08 + 0.55	5.84 + 0.13
N749 + CSGR1	**18.51**	**0.659**	**65.18**	**7.95**
	17.84 + 0.58	0.641 + 0.010	66.802 + 1.24	7.62 + 0.19
N749 + CSGR2	**18.55**	**0.674**	**67.08**	**8.40**
	18.34 + 0.44	0.659 + 0.009	66.90 + 0,74	8.07 + 0.12
N749 + CSGR3	**18.15**	**0.658**	**65.42**	**7.81**
	17.51 + 0.41	0.648 + 0.008	65.27 + 1.04	7.39 + 0.13
N749 + CSGR4	**15.72**	**0.634**	**65.83**	**6.56**
	15.10 + 0.45	0.632 + 0.005	65.95 + 0.90	6.29 + 0.22
N749 + CSGR5	**16.59**	**0.651**	**64.68**	**6.99**
	15.99 + 0.53	0.647 + 0.010	64.18 + 1.05	6.66 + 0.22

Average values and the error bars are calculated based on five cells and samples. Parameters of the best cell are highlighted in bold.

**Table 3:** EIS analysis data obtained by fitting in an equivalent circuit.

Dye	R1	R2	R3	Ʈ_e_ (ms)
N749 + CSGR1	7.138	3.165	19.38	4.46
N749 + CSGR2	6.785	3.197	20.37	4.75
N749 + CSGR3	6.763	3.328	15.28	3.89
N749 + CSGR4	7.124	3.439	12.6	2.43
N749 + CSGR5	6.393	3.538	15.64	3.08
N749	7.139	3.206	11.72	2.17

## Supplementary Materials

The Supplementary Materials are available online.

## References

[1] Badawy W.A. (2015). A review on solar cells from Si-single crystals to porous materials and quantum dots. J. Adv. Res..

[2] Matthew S.M., Niva A.R., Guillermo C.B., Richard H.F. (2018). Understanding energy loss in organic solar cells: Toward a new efficiency regime. Joule.

[3] Antonio C., Borbone F., Roberto C. (2018). Research progress on photosensitizers for DSSC. Front. Chem..

[4] Green M.A., Ho-Baillie A. (2017). Perovskite solar cells: The birth of a new era in photovoltaics. ACS Energy Lett..

[5] Ravindiran M., Praveenkumar C. (2018). Status review and the future prospects of CZTS based solar cell—A novel approach on the device structure and material modeling for CZTS based photovoltaic device. Renew. Sustain. Energy Rev..

[6] Mehmood U., Rahman S.U., Harrabi K., Hussein I.A., Reddy B.V.S. (2014). Recent advances in dye sensitized solar cells. Adv. Mater. Sci. Eng..

[7] Luceño-Sánchez J.A., Díez-Pascual A.M., Capilla R.P. (2019). Materials for photovoltaics: State of art and recent developments. Int. J. Mol. Sci..

[8] O’Regan B., Grätzel M. (1991). High-efficiency solar cell based on dye-sensitized colloidal TiO_2_ films. Nature.

[9] Yella A., Lee H.W., Tsao H.N., Yi C., Chandiran A.K., Nazeeruddin M.K. (2011). Porphyrin-sensitized solar cells with Cobalt (II/III)–based redox electrolyte exceed 12 percent efficiency. Science.

[10] Mathew S., Yella A., Gao P., Humphry-Baker R., Curchod B.F., Ashari-Astani N. (2014). Dye-sensitized solar cells with 13% efficiency achieved through the molecular engineering of porphyrin sensitizers. Nat. Chem..

[11] Kakiage K., Aoyama Y., Yano T., Oya K., Fujisawa J., Hanaya M. (2015). Highly-efficient dye-sensitized solar cells with collaborative sensitization by silyl-anchor and carboxy-anchor dyes. Chem. Commun..

[12] Pradhan S.C., Hagfeldt A., Soman S. (2018). Resurgence of DSC with copper electrolyte: A detailed investigation of interfacial charge dynamics with cobalt and iodine based electrolytes. J. Mater. Chem. A.

[13] Zhang L., Cole J.M. (2017). Dye aggregation in dye-sensitized solar cells. J. Mater. Chem. A.

[14] Naik P., Su R., Babu D.D., Ei-Shafei A., Adhkari A.V. (2017). Structurally simple D-A-type organic sensitizers for dye-sensitized solar cells: Effect of anchoring moieties on the cell performance. J. Iran. Chem. Soc..

[15] Aghazad S., Nazeeruddin M.K. (2018). Ruthenium complexes as sensitizers in dye-sensitized solar cells. Inorganics.

[16] Yum J.H., Baranoff E., Wenger S., Nazeeruddin M.K., Gratzel M. (2011). Panchromatic engineering for dye-sensitized solar cells. Energy Environ. Sci..

[17] Ganesh K., Pavan K.C.H., Paolo S., Gabriele M., Maria G.L., Olivia B., Filippo D.A., Chandrasekharam M. (2016). New terpyridine-based ruthenium complexes for dye sensitized solar cells applications. Inorg. Chim. Acta.

[18] Clifford J.N., Martinez-Ferrero E., Viterisi A., Palomares E. (2011). Sensitizer molecular structure-device efficiency relationship in dye sensitized solar cells. Chem. Soc. Rev..

[19] Salvatori P., Agrawal S., Barreddi C., Chandrasekharam M., de Borniol M., Angelis F.D. (2014). Stability of ruthenium/organic dye co-sensitized solar cells: A joint experimental and computational investigation. RSC Adv..

[20] Timofey N.C., Ekaterina A.K., Ellie T., Vadim V.P., Ludmila V.M., Neil R., Oleg A.R. (2019). [1,2,5]Thiadiazolo[3,4-d]Pyridazine as an internal acceptor in the D-A-π-A organic sensitizers for dye-sensitized solar cells. Molecules.

[21] Connell A., Holliman P.J., Davies M.L., Gwenin C.D., Weiss S., Pitak M.B., Horton P.N., Coles S.J., Cooke G. (2014). A study of dye anchoring points in half-squarylium dyes for dye-sensitized solar cells. J. Mater. Chem. A.

[22] Connell A., Holliman P.J., Jones E.W., Furnell L., Kershaw C., Davies M.L., Gwenin C.D., Pitak M.B., Coles S.J., Cooke G. (2015). Multiple linker half-squarylium dyes for dye-sensitized solar cells; are two linkers better than one?. J. Mater. Chem. A.

[23] Burke A., Schmidt-Mende L., Ito S., Gratzel M. (2007). A novel blue dye for near-IR ‘dye-sensitised’ solar cell applications. Chem. Commun..

[24] Geiger T., Kuster S., Yum J.-H., Moon S.-J., Nazeeruddin M.K., Gratzel M., Nuesch F. (2009). Molecular design of unsymmetrical squaraine dyes for high efficiency conversion of low energy photons into electrons using TiO_2_ nanocrystalline films. Adv. Funct. Mater..

[25] Yen Y.-S., Chou H.-H., Chen Y.-C., Hsu C.-Y., Lin J.T. (2012). Recent developments in molecule-based organic materials for dye-sensitized solar cells. J. Mater. Chem..

[26] Son C.H., Suresh T., Chitumalla R.K., Koyyada G., Cheruku R., Jang J., Jung J.H., Kim J.H. (2018). Synthesis and investigation of anchoring unit effect in blue-colored isoindigo-based D-A-π-A organic dyes for dye-sensitized solar cells. Jpn. J. Appl. Phys..

[27] Bao L.Q., Thogiti S., Koyyada G., Kim J.H. (2019). Synthesis and photovoltaic performance of novel ullazine-based organic dyes for dye-sensitized solar cells. Jpn. J. Appl. Phys..

[28] Lee C.P., Li C.T., Ho K.C. (2017). Use of organic materials in dye-sensitized solar cells. Mater. Today.

[29] Adewale O.A., Peter A.A. (2014). Towards the development of functionalized polypyridine ligands for Ru(II) complexes as photosensitizers in dye-sensitized solar cells (DSSCs). Molecules.

[30] Abdalhadi S.M., Connell A., Zhang X., Wiles A.A., Davies M.L., Holliman P.J., Cooke G. (2016). Convenient synthesis of EDOT-based dyes by C-H activation and their application as dyes in dye sensitized solar cells. J. Mater. Chem. A.

[31] Al-Eid M., Lim S.H., Park K.-W., Fitzpatrick B., Han C.-H., Kwak K., Hong J., Cooke G. (2014). Facile synthesis of metal-free organic dyes featuring a thienylethynyl spacer for dye sensitized solar cells. Dyes Pigments.

[32] Bella F., Gerbaldi C., Barolo C., Gratzel M. (2015). Aqueous dye-sensitized solar cells. Chem. Soc. Rev..

[33] Kurotobi K., Toude Y., Kawamoto K., Fujimori Y., Ito S., Chabera P. (2013). Highly asymmetrical porphyrins with enhanced push-pull character for dye-sensitized solar cells. Chem. A Eur. J..

[34] Han L., Islam A., Chen H., Malapaka C., Zhang S., Yang X., Yanagida M., Chiranjeevi B. (2012). High-efficiency dye-sensitized solar cell with a novel co-adsorbent. Energy Environ. Sci..

[35] Luo J., Wan Z., Jia C., Wang Y., Wu X. (2016). A co-sensitized approach to efficiently fill the absorption valley, avoid dye aggregation and reduce the charge recombination. Electrochim. Acta.

[36] Naik P., Abdellah I.M., Shakour M.A., Su R., Keremane K.S., El-Shafei A., Adhikaria A.V. (2018). Improvement in performance of N3 sensitized DSSCs with structurally simple aniline based organic co-sensitizers. Sol. Energy.

[37] Chandrasekharam M., Chiranjeevi B., Gupta K.S.V., Singh S.P., Islam A., Han L., Kantam M.L. (2012). Simple metal-free organic D-A dyes with alkoxy- or fluorine substitutions: Application in dye sensitized solar cells. J. Nanosci. Nanotechnol..

[38] Ganesh K., Shome S., Chandrasekharam M., Sharma G.D., Singh S.P. (2016). High performance dye-sensitized solar cell from a cocktail solution of a ruthenium dye and metal free organic dye. RSC Adv..

[39] Wang Q., Chen B., Wu W.J., Li X., Zhu W.H., Tian H., Xie Y.S. (2014). Efficient solar cells sensitized by porphyrins with an extended conjugation framework and a carbazole donor: From molecular design to cosensitization. Angew. Chem..

[40] Nguyen L.H., Hemant K.M., Dharani S., Sneha A.K., Sudip K.B., Kazuteru N., Gratzel M., Subodh G.M. (2012). A selective co-sensitization approach to increase photon conversion efficiency and electron lifetime in dye-sensitized solar cells. Phys. Chem. Chem. Phys..

[41] Patil D., Jadhav M., Avhad K., Chowdhury T.H., Islam A., Bedjac I., Sekar N. (2018). A new class of triphenylamine-based novel sensitizers for DSSCs: A comparative study of three different anchoring groups. New J. Chem..

[42] Chen B.-S., Chen D.-Y., Chen C.-L., Hsu C.-W., Hsu H.-C., Wu K.-L., Liu S.-H., Chou P.-T., Chi Y. (2011). Donor-acceptor dyes with fluorine substituted phenylene spacer for dye-sensitized solar cells. J. Mater. Chem..

[43] Ganesh K., Singh S.P., Bhanuprakash K., Han L., Bedja I.M., Gupta R.K., Islam A., Chandrasekharam M. (2016). Study of donor-acceptor-π-acceptor architecture sensitizers with benzothiazole acceptor for dye-sensitized solar cells. Energy Technol..

[44] Frisch M.J., Trucks G.W., Schlegel H.B., Scuseria G.E., Robb M.A., Cheeseman J.R., Scalmani G., Barone V., Mennucci B., Petersson G.A. (2009). Gaussian 09, Revision D.01.

[45] Becke A.D. (1993). Density-functional thermochemistry. III. The role of exact exchange. J. Chem. Phys..

[46] Becke A.D. (1996). Density-functional thermochemistry. IV. A new dynamical correlation functional and implications for exact-exchange mixing. J. Chem. Phys..

[47] Miertus S., Scrocco E., Tomasi J. (1981). Electrostatic interaction of a solute with a continuum. A direct utilizaion of AB initio molecular potentials for the prevision of solvent effects. Chem. Phys..

[48] Cossi M., Barone V., Cammi R., Tomasi J. (1996). Ab initio study of solvated molecules: A new implementation of the polarizable continuum model. Chem. Phys. Lett..

[49] Liu B., Li W., Wang B., Li X., Liu Q., Naruta Y., Zhu W. (2013). Influence of different anchoring groups in indoline dyes for dye-sensitized solar cells: Electron injection, impedance and charge recombination. J. Power Sources.

[50] Babu D.D., Su R., El-Shafei A., Adhikari A.V. (2016). New indole based co-sensitizers for dye sensitized solar cells exceeding 10% efficiency. RSC Adv..

[51] Bonomo M., Barbero N., Naponiello G., Giordano M., Dini D., Barolo C. (2019). Sodium hydroxide pretreatment as an effective approach to reduce the dye/holes recombination reaction in P-type DSCs. Front. Chem..

