# Supplementary material for: A New Series of EDOT Based Co-Sensitizers for Enhanced Efficiency of Cocktail DSSC: A Comparative Study of Two Different Anchoring Groups
Source: Molecules. 2019 Sep 30;24(19):3554. doi: 10.3390/molecules24193554 (PMC6803997; doi:10.3390/molecules24193554)
Supplement: Supplementary file 1 [file molecules-24-03554-s001.pdf]

**A new series of EDOT based co-sensitizers for enhanced efficiency of cocktail DSSC: A comparative study of two different anchoring groups**

Ganesh Koyyada[a], Ramesh Kumar Chitumalla[b], Suresh Thogiti[a], Jae Hong Kim[a], Joonkyung Jang[b], Malapaka Chandrasekharama[c,d]*, Jae Hak Jung [a,]*

[a]Department of Chemical Engineering, Yeungnam University, 214-1, Dae-hakro 280, Gyeongsan, Gyeongbuk, 712-749, South Korea.

[b]Department of Nanoenergy Engineering, Pusan National University, Busan 46241, South Korea

[c]Inorganic and Physical Chemistry Division, CSIR-Indian Institute of Chemical Technology, Tarnaka, Hyderabad 500007, India.

[d]Academy of Scientific & Innovative Research (AcSIR), CSIR-IICT

E-mail: jhjung@ynu.ac.kr, csmalapaka@iict.res.in,

**1.1. Synthesis:**

**2,3-dihydrothieno[3,4-b][1,4]dioxine-5-carbaldehyde (1) [1]:**

3,4-Ethylenedioxythiophene (2 mL, 18 mmol) was dissolved in dry N N-Dimethylformamide (DMF) (10 mL, 126 mmol), the mixture was cooled to -10 °C and $POCl_3$ (1.76 mL, 18 mmol) was added slowly dropwise over  min. The reaction mixture was then allowed to reach room temperature then stirred for an additional hour. The reaction was poured into an ice bath and neutralized using a basic aqueous solution. The product, in the form of white needles, was filtered and dried, yielding a quantitative yield (3.06 g). $^1$H NMR (300 MHz, $CDCl_3$ , δppm ): 9.83 (s, 1H), 6.74 (s, 1H), 4.31 (d, 2H), 4.21 (d, 2H). $^{13}$C NMR (75 MHz, $CDCl_3$ , δppm ): 180.0, 141.7, 110.8, 110.7, 65.2, 64.3.

**7-bromo-2,3-dihydrothieno[3,4-b][1,4]dioxine-5-carbaldehyde [2]:**

Compound 1 (4.04 g, 23.7 mmol) was suspended in dry acetonitrile (100 mL) and cooled to 0 °C. NBS (4.36 g, 26.0 mmol), 1.1 equiv, was added and the mixture was stirred for 60 h at room temperature, shielded from light and under nitrogen. The color changed from yellow to purple. The mixture was transferred with 150 mL of ethyl acetate to a separation funnel, washed with 10% aqueous $Na_2CO_3$ (2×200 mL), saturated $Na_2S_2O_3$ (2×200 mL) and water (2×200 mL), dried with MgSO4, and evaporated in vacuo. Recrystallization twice from

ethanol (60 mL) yielded 5.35 g (91%) of the bromide as yellow needles. $^{1}$H NMR (300 MHz, $CDCl_3$ δ ppm ): 9.83 (s, 1H), 4.36 (m, 4H); $^{13}$C NMR (75 MHz, $CDCl_3$ δ ppm):179.0, 147.9, 140.4, 118.8, 102.0, 65.5, 65.1.

**7-(2,3,4-trimethoxyphenyl)-2,3-dihydrothieno[3,4-b][1,4]dioxine-5-carbaldehyde (3a) [3]:** 50 mL Schlenk tube was charged with (5-formylfuran-2-yl)boronic acid (0.140 g, 1 mmol) and [$Pd(PPh_3)_4$] (0.113 g, 0.01 mmol). Dimethoxy ethane (8 mL) and 2m aqueous sodium carbonate (2 mL) were added, and the tube was purged with argon gas with five evacuate/refill cycles. Compound 5a (0.534 g, 1 mmol) was subsequently added as a neat liquid. The tube was sealed and heated at 908C for 18 h. Upon cooling to ambient temperature, the organic compounds were extracted into dichloromethane (3× 30 mL) from water (30 mL). The combined organic layers were washed with water (1×30 mL) and brine (1×30 mL), dried over $Na_2SO_4$, filtered, and the solvent was removed under reduced pressure. The crude product was preadsorbed onto silica gel and purified by column chromatography (9:1 hexane/ethyl acetate) to give 6a (0.395 g, 74%).$^{1}$HNMR (300 MHz, $CDCl_3$, δppm ) 10.11 (s, 1H), 7.86 (d, 1H), 6.51 9d, 1H) 4.32 (m, 4H), 3.92 (s, 3H), 3.84 (s, 6H).

**7-(2,4-dibutoxyphenyl)-2,3-dihydrothieno[3,4-b][1,4]dioxine-5-carbaldehyde (3b):**

Compound 3b was synthesized by following the same procedure as that employed for the synthesis of 3a by using (2,4-dibutoxyphenyl)boronic acid (0.xx g, 1 mmol), [$Pd(PPh_3)_4$] (0.xx g, 0.1 mmol), dimethoxy ethane (8mL), a 2m aqueous solution of sodium carbonate as a yellow liquid (0.384 g, 72%). $^{1}$HNMR (300 MHz, $CDCl_3$, δppm ) 10.12 (s, 1H), 7.81 (d, 1H), 7.01 (d, 1H), 6.84 (s, 1H), 4.26 (m, 8H), 1.89 (m, 4H), 1.47 (m, 4H), 0.91 (t, 6H)

**7-(2,4-difluorophenyl)-2,3-dihydrothieno[3,4-b][1,4]dioxine-5-carbaldehyde (3c):**

Compound 3b was synthesized by following the same procedure as that employed for the synthesis of 3a by using (2,4-difluorophenyl)boronic acid (0.xx g, 1 mmol), [$Pd(PPh_3)_4$] (0.xx g, 0.1 mmol), dimethoxy ethane (8mL), a 2m aqueous solution of sodium carbonate as a yellow liquid (0.384 g, 72%). $^{1}$HNMR (300 MHz, $CDCl_3$, δppm ) 10.12 (s, 1H), 7.71 (d, 1H), 7.31 (s, 1 H), 7.16 (d, 1H), 4.32 (m, 4H).

## 1.2. Solar cell fabrication and Photovoltaic characterization

The transparent conducting glasses (fluorine-doped tin oxide (FTO)) were cleaned carefully with ethanol, D.I water and acetone in anultra-sonication process, respectively. The

transparent $TiO_2$ pastes (20-30 nm, Dyesol Ltd.) were coated on cleaned FTO glasses (Pilkington, 15 Ω/cm$^2$) using the doctor blade technique, followed by calcination for 30 min at 450 $^0$C. The $TiO_2$scattering layer (200 nm, Dyesol Ltd.) consists of rutile $TiO_2$ was deposited on the transparent mesoporous $TiO_2$ films, followed by sintering at 450 $^0$C for 30 min. Two layers of $TiO_2$ films were dipped in a 40 mM aqueous solution of $TiCl_4$ at 70 $^0$C for 30 min and then sintered at 450 $^0$C for 30 min. The $TiO_2$ thin films were immersed in the dye solution for 24 hours in dark at 25 $^0$C. where dye solution was prepared using 0.1 mM of CSGR dyes and 0.2 mM N749 dye in tert-butanol and acetonitrile mixed solvent (1 : 1). After 24 hours soaking the residual dye was rinsed with acetonitrile and dried. The platinum catalyst was coated on the FTO glasses using the doctor blade technique by $H_2PtCl_6$ solution, followed by pyrolysis at 450 $^0$C for 30 min to prepare the counter electrodes. The working electrode and Pt counter electrodes were assembled into a sealed sandwich cell with a 60 mm thick thermal adhesive film (Surlyn film, Dupont), which was then introduced with a liquid electrolyte solution contained 1-butyl-3- methylimidazolium iodide (0.7 M), lithium iodide (LiI, 0.2 M), iodine ($I_2$, 0.05 M), and t-butylpyridine (TBP, 0.5 M) in acetonitrile/ valeronitrile (85:15, v/v) through pre-drilled two holes on the counter electrode.

The synthesized dyes photo-current density-voltage (J-V) characteristics were measured under AM 1.5 irradiation with an irradiance of 100 mW/cm$^2$ (PEC-L11, Peccell Technologies, Inc.). The incident monochromatic photon-to-current efficiencies (IPCEs) were measured using IPCE measurement instrument (PEC-S20, Peccell Technologies, Inc.) as a function of wavelength. Electrochemical impedance spectroscopy (EIS) was recorded using computer-controlled potentiostat (IVIUMSTAT, IVIUM) software at the open circuit voltage with a 10 mV of amplitude and an AC frequency range between 100 kHz and 0.1 Hz (PEC-L11, Peccell Technologies, Inc.).

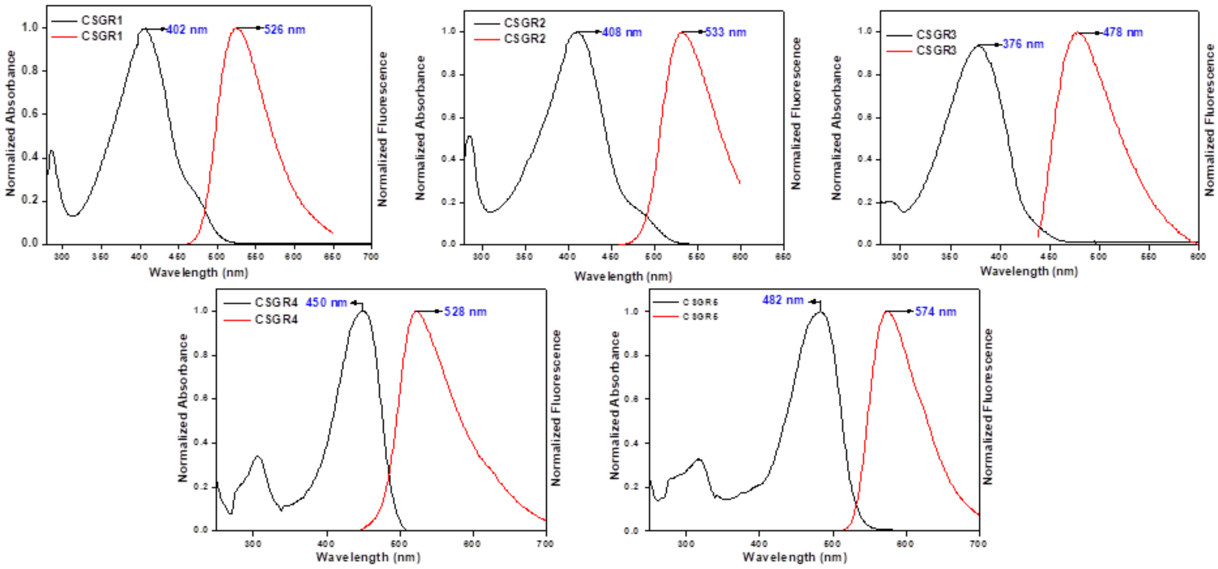


**Figure S1.** Absorption and fluorescence intercept plots.

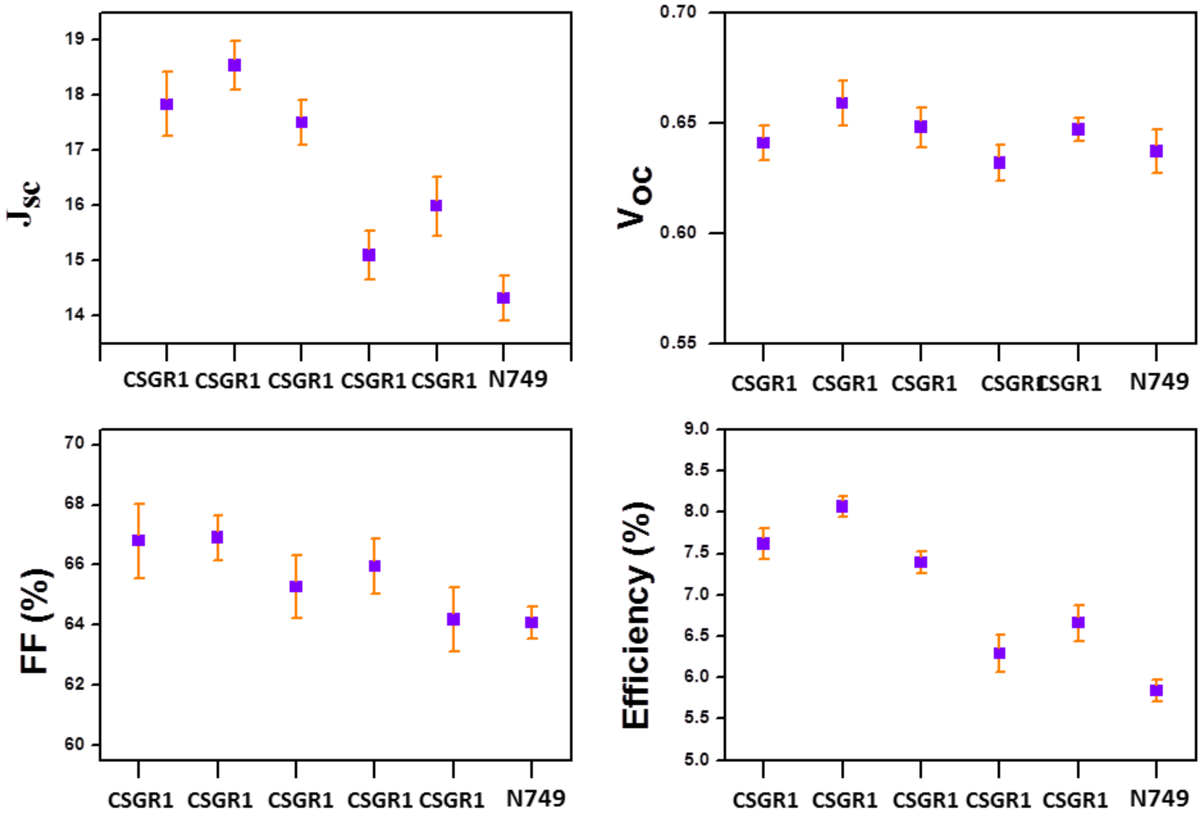


**Figure S2.** Standard error analysis of photovoltaic parameters of co-sensitized DSSCs of CSGR and N749 dyes

**References:**


1. L. Cai, H. N. Tsao, W. Zhang, L. Wang, Z. Xue, M. Grätzel, B.Liu, Adv. Energy Mater. 3, 200 (2012).
2. M. Jessing, M. Brandt, K. J. Jensen, J. B. Christensen, U. Boas, J. Org. Chem. 71, 6734 (2006).
3. M. Chandrasekharam, B. Chiranjeev, K. S. V. Gupta1, S. P. Singh, A. Islam, L. Han, and M. L. Kantam, Journal of Nanoscience and Nanotechnology 12, 4489 (2012).
